# Microplastic pollution in the marine environment: Sources, impacts, and degradation

**DOI:** 10.5455/javar.2025.l893

**Published:** 2025-03-25

**Authors:** Osamah A. Ahmad, Mamdoh T. Jamal, Hamdah S. Almalki, Abeer H. Alzahrani, Amirah S. Alatawi, Md Fazlul Haque

**Affiliations:** 1 Department of Marine Biology, Faculty of Marine Sciences, King Abdulaziz University, Jeddah, Saudi Arabia; 2 Department of Animal Biology, Faculty of Sciences, King Abdulaziz University, Jeddah, Saudi Arabia; 3 Department of Zoology, Faculty of Biological Sciences, University of Rajshahi, Rajshahi, Bangladesh

**Keywords:** Degradation, ecotoxicity, marine organisms, pollution, microplastics

## Abstract

Microplastics (MPs) are tiny particles derived from plastic, and their high fragmentation poses a significant threat to the marine environment. MPs can originate from various sources, such as primary or secondary sources as well as internal or external sources. However, in marine environments, MPs mainly enter from external sources, such as plastic waste, debris from land-based activities, tourism, shipping, and atmospheric deposition. MP accumulation in marine ecosystems is also influenced by the use of plastic equipment, aquaculture feed, health products, and particular environmental conditions. Understanding the ecotoxicological effects of environmentally relevant concentrations of MPs on the health of marine organisms is crucial. The effects of MPs on the health of marine organisms are well reported by different studies, highlighting their interactions with a wide range of marine life, including plankton, invertebrates, crustaceans, corals, seagrass, fish, and even humans. Thus, MPs have been reported as a notorious pollutant because of their deleterious impacts on the morphology, physiology, immunology, behavior, reproduction, and heredity of marine organisms. Moreover, most MPs are slowly degradable, and degradation is dependent on various biotic and abiotic factors, resulting in continuous accumulation in the marine environment. This review provides an in-depth explanation of the sources of MPs, along with their impacts on marine organisms, ecosystems, aquaculture, and human health. In addition, it will discuss the degradation of MPs in the marine environment to explore the potential strategies for reducing the harmful impacts of MPs.

## Introduction

Plastics are materials made from various synthetic and semisynthetic organic ingredients that are initially soft and eventually become hard or slightly flexible [1]. Plastics find widespread use in everyday life due to their lightweight, durability, low cost, and excellent ductility [2]. Over the past 70 years, plastic production has skyrocketed, reaching 368 million tons in 2019, marking the era as a plastic world [3]. The global production of plastics has surged from 1.0 million tons in the 1950s to 7.0 billion tons annually by 2020, with an annual increase of 10%. By 2025, production is expected to rise to 15.7 billion tons [4].

In comparison to other regions, plastic consumption in Asian countries is relatively lower. China is the largest producer, contributing 59.08 million tons, followed by the United States with 37.83 million tons and Germany with 14.48 million tons. Other countries such as Brazil, Japan, Pakistan, and Nigeria are also major contributors to global plastic production, with India ranked 15th [5]. Moreover, various other pollutants, including petroleum hydrocarbons, textile dyes, and industrial effluents, can be degraded by microorganisms [6–12]. In contrast, plastics are highly resistant to microbial biodegradation, leading to their prolonged persistence in the environment and posing a significant environmental challenge [13,14]. Improper waste management and disposal lead to large quantities of plastic waste entering the environment, contributing to severe pollution. Once in the environment, plastics gradually break down into smaller particles, known as microplastics, which have become a global environmental issue [15].

Although plastic materials are relatively new materials, they cover almost all aquatic environments, resulting in the reduction of sources for safe and pollutant-free water [16,17]. On the other hand, the global water demand nearly doubles every 20 years due to population growth and industrial expansion [18]. This study highlights the critical role of plastic as a pollutant carrier, impacting both human and marine life. Microplastics (MPs) often contain synthetic polymers that do not break down, such as polyethylene (PE), polypropylene (PP), polyamide (PA), polyvinyl chloride (PVC), polystyrene (PS), and PE terephthalate (PET) [19,20]. These polymers are frequently present as the main ingredients in MPs, which come in varying sizes, such as 10, 5, 2, and 1 mm in diameter [5,19–21].

Besides packaging, fishing equipment such as fish nets, ropes, and fishing lines are often made from polyolefins. These plastics absorb toxic pollutants such as polychlorinated biphenyls (PCBs) and dichlorodiphenyltrichloroethane (DDT) more readily [22]. Moreover, MPs also act as carriers for heavy metals in the environment, facilitating their transport [23]. MPs interact with heavy metals such as cadmium (Cd), lead (Pb), and arsenic (As), which are released into the environment in vast quantities through industrial activities [24]. These interactions can lead to synergistic or antagonistic effects on plants (Table 1). Exposure to both MPs and heavy metals together increases the likelihood of plants becoming toxic, according to recent research [23,24]. This is because MPs and heavy metals can disrupt various biological and physiological processes. Furthermore, experts declare MPs to be among the world’s most pervasive and dangerous marine pollutants. Hence, Goal 14 of the United Nations Sustainable Development Goals, which aims to conserve and sustainably use the oceans, seas, and marine resources for sustainable development, is mainly focused on protecting and conserving the marine environment from MPs [25]. Hence, research on plastic pollution, especially on pollution of aquatic environments with microplastics and nanoplastics, has become a popular topic for scientists to ensure a safe and inexpensive water supply as well as for the preservation of aquatic environments.

The marine environment encompassing seafloor sediment, deep waters, shorelines, and oceans is a primary location for microplastic accumulation [26]. Coral reef areas, in particular, are prone to microplastic buildup [27,28]. Due to their ultrasmall size, MPs and nanoplastics (Nps) are easily ingested by marine organisms. When smaller marine species, such as zooplankton, ingest these MPs, they are transferred to secondary consumers, like large fish, and ultimately to tertiary consumers. This procedure disrupts the food chain and food web [29]. Studies have also assessed the levels of ingested plastic in various fish species collected from the North Sea at different locations [30].

MPs have been shown to have a range of harmful effects on marine organisms, including genotoxicity, neurotoxicity, reduced feeding activity, growth delays, and decreased reproductive fitness [31–35] (Table 1). Marine species are also at risk of eating more pollutants and dangerous substances when MPs get into the food chain. For example, toxic heavy metals that stick to the surface of MPs or are built into their structure [36]. These contaminated MPs pose significant risks to both human and marine animal health. Hydrophobic pollutants in seawater tend to attach to plastic debris under typical environmental conditions [37]. The impacts of these pollutants are especially concerning because they are toxic, bioaccumulative, and persistent. Plastics transport harmful compounds and enhance their longevity in the environment [38].

Plastic waste has been widely reported in marine environments, including remote locations like the Pacific islands [1,39]. Key sources of plastic waste entering the ocean include urban, industrial, boating, shipping, fishing, and aquaculture activities [1]. Studies worldwide have shown that plastic debris is prevalent in benthic, pelagic, and coastal areas of virtually all marine ecosystems [40]. Because plastic trash is found in large amounts in marine ecosystems and has unique chemical and structural properties, it can get into a wide range of invertebrate and vertebrate species and have several harmful effects (Table 1). Moreover, plastics have the potential to transport both organic and inorganic hazardous chemicals from land to marine environments and ultimately to humans via the food chain, raising significant global concerns [13].

### Microplastics

MPs are small plastic particles, such as pieces, fibers, or beads, that measure smaller than 5 mm in their longest dimension. These particles can be classified into two types: primary and secondary MPs [5,41]. They also vary in terms of size, shape, polymer type, and color [42,43]. Primary MPs are tiny or microscopic in structure and are directly or indirectly released into the environment through sources such as sewage discharges, household and industrial effluents, spills, and wastewater [44]. Common forms of primary MPs include pellets, films, fragments, and spheres. On the other hand, secondary MPs are made when larger pieces of plastic are broken down by ultraviolet (UV) photo-oxidation, mechanical degradation, and microbial degradation [5].

### Sources of microplastics

MPs have been detected in diverse environments across the globe, including terrestrial ecosystems (such as land, islands, cities, and beaches), aquatic ecosystems (including marine, river, lake, reservoir, and pond environments), and the atmosphere [13,21,40,45,46]. Due to improper disposal, an estimated 4.8–12 million tons of plastic waste enter the ocean annually [40]. Hazardous pieces of plastic can be found in both land and water ecosystems because of human activities, such as those that happen in homes, factories, and along the coast [47,48]. In aquatic ecosystems, MPs primarily originate from domestic runoffs, which contain microbeads and fragments from consumer products like cosmetics, as well as from the breakdown of larger plastic waste [42]. Key contributors to microplastic pollution in marine environments include general littering, plastic waste mismanagement, tires, synthetic textiles, marine coatings, road markings, personal care products, plastic pellets, city dust, and the release of wastewater from sewage treatment plants [29,49–51]. Marine litter largely arises from the improper disposal of refuse, which is transferred either directly or indirectly to seas and oceans [52].

In aquaculture environments, MPs primarily originate from land-based plastic waste, including waste discarded from tourism, shipping, transportation, fisheries, and atmospheric deposition [40,45,53–55]. Land-based plastic litter is the primary source of MPs in the aquatic environment, contributing to 80% of marine plastic pollution. This category includes primary MPs found in cosmetics and air-blasting materials, improperly disposed plastics, and leachates from waste sites. Nearly half of the global population resides within 50 miles (ca. 80 km) of the coast, making it highly likely for these plastics to enter the marine ecosystem through rivers, wastewater systems, or wind-driven transport [52].

Shipping transportation also plays a significant role in marine microplastic pollution, with plastic waste discarded from ships contributing to the problem. The incomplete burning of plastic materials during shipping processes releases plastic particles into the atmosphere and water [40,56]. This phenomenon occurs when plastic materials (e.g., waste or packaging) are improperly incinerated or burned at low temperatures during shipping activities, either on ships or at ports. Incomplete combustion leads to the release of small plastic particles and toxic by-products into air and water [56]. This process primarily disperses microplastics and airborne pollutants, which can settle into marine environments or spread over long distances, affecting ecosystems far from the source [40].

On the other hand, shipping accidents such as container spills, shipwrecks, or collisions can release large quantities of plastic products or raw plastic materials directly into the ocean [40,56]. Thus, the spilled materials enter the marine environment as large pieces of plastic, leading to localized and immediate pollution, with concentrated impacts in the vicinity of the spill. However, the large pieces of plastic may fragment into microplastics over time due to physical, chemical, and biological processes such as wave action and UV exposure [56]. Thus, these plastics may travel with ocean currents and contribute to the global microplastic problem [40,56].

Fishing and aquaculture activities are inherently linked to plastic use. Many fishing tools, such as nets, lines, buckets, and other equipment, are made from or contain plastic. Over time, as these materials are used and discarded, they release MPs into surrounding environments [40]. Additionally, MPs in the atmosphere are deposited on land and in aquatic environments due to gravity and weather conditions [40].

These microscopic plastics are ingested by various marine organisms, including corals, plankton, marine invertebrates, fish, and whales, and they can travel up the food chain. The ingestion of biodegradable plastics poses a direct threat to marine species and indirectly interacts with other marine pollutants in complex ways that can amplify or alter their environmental impact [52]. These interactions primarily occur through physical, chemical, and biological processes, influencing the overall toxicity and bioavailability of pollutants in the marine ecosystem [52].

### Primary sources of microplastics

The primary sources of MPs are synthetic polymeric materials such as PVC, PET, polymethyl methacrylate (PMMA), and acrylic, which are used in various residential and industrial applications [57]. These materials give rise to MPs with diverse shapes, sizes, and chemical compositions, depending on their intended uses. For example, MPs in personal care products typically have sizes smaller than 0.5 mm, while PS spheres are usually smaller than 2 mm, and PE and PP granules are generally smaller than 5 mm [58]. One of the main sources of MPs is plastic pellets, also known as nibs, which are pelletized resins with a length of 2–5 mm. These pellets serve as precursors in the production of various plastic-based materials [59].

Additional primary sources of MPs include microbeads used in personal care products, where they function as exfoliating agents or abrasives in cosmetics such as face wash, toothpaste, nail polish, hair products, and deodorants [60,61]. Glitter, another example, is a type of microplastic used in fabrics and personal care products. Additionally, MPs from primary sources are used in air-blasting equipment [62]. While microbeads in personal care products are a direct source of primary MPs, secondary MPs result from the degradation of larger plastic items, such as macroplastics, which break down when exposed to environmental factors such as high temperatures, sunlight, and strong winds [61]. These environmental factors often act together in a synergistic manner. For instance, plastics exposed to sunlight in seawater may undergo photodegradation, oxidation, and physical fragmentation simultaneously, leading to accelerated production of secondary MPs [61].

### Secondary sources of microplastics

Secondary sources of MPs are generated in the environment as larger plastic materials (macroplastics) break down into tiny fragments through various degradation processes. These processes include photodegradation, thermal degradation, chemical degradation, biodegradation, weathering, corrosion, and mechanical erosion caused by external forces such as wind and water [63]. Wastewater treatment plants (WWTPs) also contribute significantly to microplastic pollution, as wastewater contains numerous polymers from sources such as cosmetics, facial scrubs, and textiles that are inadequately filtered during treatment [64,65]. However, the development of some advanced technologies and the optimization of the existing processes of WWTPs can adequately filter MPs from wastewater [64,65]. For example, ultrafiltration, nanofiltration, or reverse osmosis membranes can capture MPs based on size exclusion [64]. Similarly, adding coagulants (e.g., aluminum or iron salts) and flocculants promotes the aggregation of MPs into larger particles, making them easier to remove in sedimentation or filtration stages [65]. Additionally, MPs from food waste, landfill leachate, and personal protective equipment (PPE) used extensively during the COVID-19 pandemic serve as other secondary sources of MPs [63]. During the COVID-19 pandemic, mandatory face masks, often made from nonwoven polyester, PP, PS, and PE materials, were the sources of MPs in the environment [66,67].

Effluents from WWTPs are among the most significant secondary sources of MPs entering marine environments [53]. MPs can be released during the early stages of water treatment. Due to their small size, they can pass through porous filters and reach secondary treatment units, such as biological treatment units. Typically, influents contain between 1 and 10,000 microplastic particles per liter, whereas treated effluents may contain anywhere from 0 to 450 particles per liter, depending on the treatment’s effectiveness [68]. Furthermore, microfibers from polyester, acrylic, and PA materials have been detected in WWTP effluents [69], highlighting the limitations of these treatment plants in removing microplastic contamination [63].

A prominent example of this secondary source is microplastic particles released into the environment through the washing of fabrics and textiles [70]. It is estimated that laundering clothes contributes nearly 500,000 tons of MPs to marine environments annually [51]. In addition to fabric erosion, household items such as toys, plastic goods, single-use plastics, fishing nets, ropes, and automobile tires also degrade over time, releasing MPs [63].

Plastic dust also enters the environment through activities such as plastic production, plastic waste incineration, traffic emissions, road deterioration, and urban mining operations [71]. The wind transports airborne plastic dust, which can settle indoors, particularly in homes and schools. Common household items made from plastic, such as food packaging, clothing, and furniture, are primary sources of airborne MPs in residential environments.

Landfills, which hold approximately 50% of all plastics produced globally, are another key source of secondary MPs. Macroplastics disposed of in landfills undergo complex biochemical processes and physical alterations that result in the formation of MPs [72]. These MPs are often irregular and jagged in shape, indicating that plastic degradation is a major source of secondary MPs in landfills [73]. Furthermore, landfills receive primary MPs through various channels, such as influent sludge streams. The presence of MPs in sludge ranges from 6.44 × 10^8^ to 1.67 × 10^12^ MPs per 1,000 t sludge [74]. Leachate from landfills can also release MPs, which may ultimately be transported to nearby water bodies, sediments, and the aquatic food chain [73]. A study reveals that minute MPs (<50 µm) can account for over 50% in landfill leachate at the following concentrations: 235.4 ± 17.1 item/l and 11.4 ± 0.8 µg/l [75].

**Figure 1.**
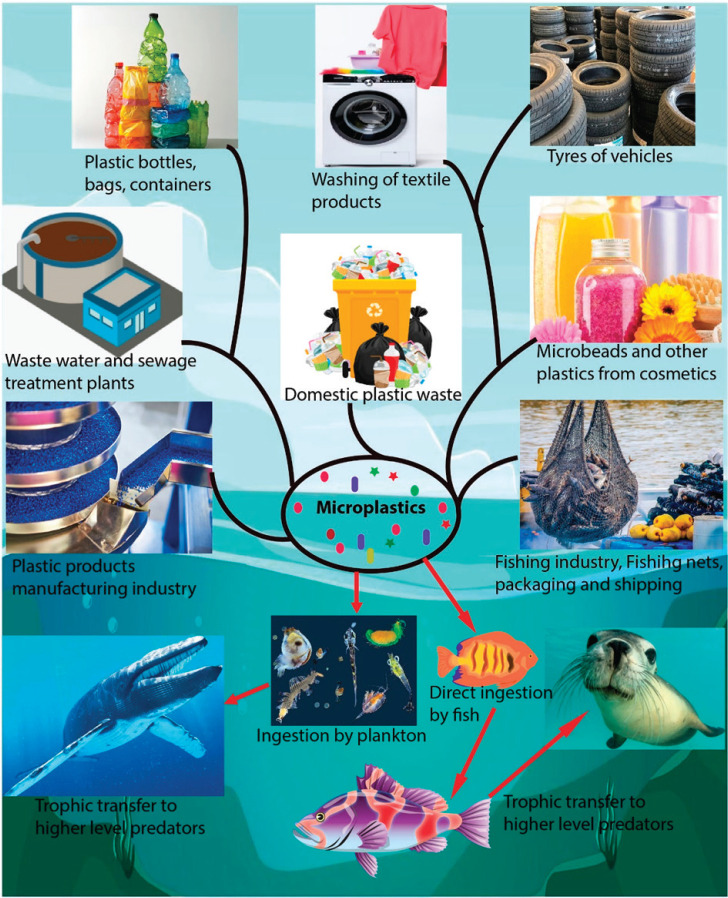
Sources and trophic transfer of microplastics in the marine environment.

In the food industry, microplastic contamination may arise from the extensive use of plastics in food manufacturing, processing, and packaging. Studies have shown that fresh fruits tend to have higher levels of MPs than vegetables, primarily due to their exposed surfaces and growth patterns [76]. For example, smooth or waxy outer surfaces of fruits often can facilitate the adherence of MPs, which are challenging to remove completely, even with thorough washing [77]. Improper waste management may lead to the release of MPs from contaminated food or food waste into the marine environment [78]. MPs can also move up the food chain through trophic transfer, potentially ending up in food or food waste [79]. For example, MPs are often mistakenly ingested by plankton as food particles, and then, these plankton are consumed by small fishes, such as anchovies or sardines, and marine mammals, such as baleen whales [79]. Similarly, larger predators such as mackerel, tuna, or seabirds prey on smaller fish that have already ingested microplastics, accumulating these particles in their digestive systems (Fig. 1) [79]. However, further research is needed to better understand the role of food waste in contributing to secondary MPs in marine environments, as knowledge in this area is still limited [78].

### Microplastics transport routes in the marine environment

MPs enter the marine environment through four primary routes [55]. One major pathway is through the deposition of plastic litter onto terrestrial areas, from where it can migrate via surface runoff caused by rainfall. Notably, most of the MPs entering the marine environment are transported by surface runoff, with wind also playing a key role by carrying plastic waste from land to the sea through atmospheric currents [55,80]. Other main sources of MP transportation into marine ecosystems are wastewater streams and the direct disposal of plastic waste into the oceans [55,80]. One notable activity contributing to the direct discharge of plastic waste is washing clothes in rivers, particularly in rural areas [63]. Once these particles enter rivers, they are subject to transport processes similar to other sediments, such as sand and silt. The faster the river flows, the greater the energy available to entrain and transport a larger volume of particles [54]. Additionally, coastal tourism activities, such as fishing and recreational pursuits, contribute to MP pollution through single-use plastic cups, litter, and commercial fishing gear like nets [81].

The density of MP polymers relative to seawater (typically ~1.02–1.03 gm/cm³) determines whether it floats, remains suspended, or sinks, significantly influencing its transport and accumulation in marine environments [54,82]. MP polymers with densities lower than seawater, such as PE (0.91–0.96 gm/cm³) and PP (0.85–0.92 gm/cm³), float on the surface of water [54,82]. These plastics can be transported over long distances by ocean currents and wind, contributing to the formation of garbage patches in the open ocean. MPs with densities close to seawater, such as nylon or some blends of PET, may become neutrally buoyant, remaining suspended in the water column [54,82]. They can be carried by currents to distant locations or ingested by marine organisms. However, MPs denser than seawater, such as PVC (1.16–1.58 gm/cm³) and PS (1.05–1.07 gm/cm³), tend to sink, accumulating in sediments and on the seabed [54,82]. These plastics can persist in deep marine environments, impacting benthic ecosystems [54,82].

**Table 1.** Deleterious effects of microplastics on diverse types of marine animals.

Effects	Mechanisms	Marine animals affected: example with reference
Physical ingestion: Blockage and damage of digestive systems, leading to starvation and reduced growth	MPs accumulate in the digestive tract, creating blockages or damaging internal tissues	Zooplankton: Copepod (*Neocalanus cristatus*) [87,88]; Crustacean: Norway lobster (*Nephrops norvegicus*) [126]; Fish: salmon-bass (*Argyrosomus regius*) [127]; Seabird: Little-black cormorant (*Phalacrocorax sulcirostris*) [128]; Sea turtle: Loggerhead turtles (*Caretta caretta*) [129])
Energy depletion: Reduced energy stores due to false satiation	Organisms sense “fullness” after ingesting indigestible MPs, leading to a lack of appetite	Coral: Stony cup coral (*Astroides* *calycularis*) [102]; Crustacean: Crab (*Carcinus maenas*) [130]; Mollusk: Pearl Oyster (*Pinctada margaritifera*) [131], Thick shell mussel (*Mytilus coruscus*) [132]; Fish: Zebrafish (*Danio rerio*) [133], California Grunion (*Leuresthes tenuis*) [134]; Sea turtle: Green turtles (*Chelonia mydas*) [135];
Toxicity: Chemical toxicity from additives (e.g., phthalates, bisphenols, and dyes)	MPs release harmful additives or adsorbed pollutants like heavy metals and PAHs, disrupting various physiological processes	Coral: Button Polyps (*Protopalythoa* sp.) [136]; Mollusk: Mussel [137], Webfoot octopus (*Amphioctopus fangsiao*) [138]; Echinoderm: Sea cucumber (*Apostichopus* *japonicus*) [139], Sea urchins [137]; Fish: European seabass (*Dicentrarchus labrax*) [140], Atlantic cod (*Gadus morhua*) [141]; Spotted sea bass (*Lateolabrax maculatus*) [142].
Bioaccumulation: Bioaccumulation of toxins through the food chain	MPs adsorb persistent organic pollutants, transferring toxins through trophic levels	Crustacean: Predatory marine crab (*Charybdis japonica*) [143]; Fish: Zebrafish (*Danio rerio*) [144], Yellowfin tuna (*Thunnus albacares*) [145]; Seabird: Galápagos penguin [146].
Habitat disruption: Smothering of benthic habitats and coral reefs	MPs settle on seabed and reefs, physically blocking light and oxygen flow	Coral: Branching coral (*Pocillopora acuta*) [147], Benthic invertebrates: Tubeworm (*Spirobranchus triqueter*) [148].
Reproductive effects: Impaired reproduction, reduced fertility, and abnormal development	MPs interfere with gamete quality, embryo development, and larval growth	Crustacean: Marine copepod (*Tigriopus japonicus*) [149]; Mollusk: Pacific oyster (*Crassostrea gigas*) [150], Mediterranean mussel (*Mytilus galloprovincialis*) [151]; Fish: Marine medaka (*Oryzias melastigma*) [152], Zebrafish (*Danio rerio*) [32,153]
Immune suppression: Oxidative stress leading to reduced immune responses	Microplastic-induced stress lowers the ability to fight infections, increasing susceptibility to disease	Crustacean: Pacific White Shrimp (*Litopenaeus vannamei*) [154]; Mollusk: Blood clam (*Tegillarca* *granosa*) [155,156]; Fish: Barramundi (*Lates* *calcarifer*) [157], Spotted sea bass (*Lateolabrax maculatus*) [142], Largemouth bass (*Micropterus salmoides*) [158]
Behavioral changes: Altered feeding, movement, or predator avoidance	Neurotoxic chemicals disrupt sensory and motor functions, affecting natural behaviors	Crustacean: Mysid shrimp (*Neomysis japonica*) [159]; Fish: Zebrafish (*Danio rerio*) [32], Yellowtail kingfish (*Seriola lalandi*)[160]; Sea turtle: Loggerhead sea turtle (*Caretta caretta*) [161]
Growth inhibition: Reduced growth and developmental delays	Energy is diverted from growth to counteract stress caused by MPs	Crustacean: Marine copepod (*Tigriopus japonicus*) [149]; Echinoderm: Sea urchin (*Paracentrotus lividus*) [162], Sea cucumber (*Apostichopus japonicus*) [139]; Fish: Marine medaka (*Oryzias melastigma*) [163]
Mortality: Increased mortality due to ingestion, starvation, or toxicity	Severe blockages, toxic overload, or nutritional deficiencies lead to death	Crustacean: Marine copepod (*Acartia tonsa*) [164], Fish: Salmonid fish [165]; Sea turtle: Sea turtles [166]; Seabird: Albatross (*Diomedea*) [167,168]; Aquatic mammal: Common bottlenose dolphin [169]
Genotoxicity: DNA damage and genetic mutations	Oxidative stress from MPs or associated toxins causes DNA fragmentation and mutations	Mollusk: Mediterranean mussel (*Mytilus galloprovincialis*) [170]; Echinoderm: Sand Dollar (*Scaphechinus mirabilis*) [171], Sea urchins (*Paracentrotus lividus*) [172]; Fish: Zebrafish (*Danio rerio*) [35], Common marine fishes [173]
Endocrine disruption: Hormonal imbalances affecting reproduction and growth	Additives like bisphenol A (BPA) mimic hormones, disrupting signaling pathways	Mollusk: Blood clam (*Tegillarca* *granosa*) [156]; Echinoderm: Sea cucumber (*Apostichopus japonicus*) [174]; Fish: Atlantic Cod (*Gadus morhua*) [175], Coral reef fish [176]
Pathogen transport: Vectors for pathogens like *Vibrio* spp.	MPs act as floating carriers for harmful bacteria, increasing infection risks	Crustacean: Pacific white shrimp (*Litopenaeus vannamei*) [177,178]; Mollusk: Clams (*Ruditapes* spp) [177–179]; Echinoderm: Sea cucumber (*Apostichopus japonicus*) [177,178]; Fish: Red sea bream (*Pargus major*) [177,178]
Invasive species: Spread of invasive species	MPs serve as rafts for invasive species, enabling them to colonize new areas	Coral: Sun coral (*Tubastraea* spp.) [180]; Mollusk: Charru mussel (*Mytella strigata*) [181], Girdled horn snail (*Pirenella cingulata*) [181]; Tunicate: Marine vomit (*Didemnum vexillum*) [182]
Trophic transfer: Transfer of MPs and associated toxins through food webs	Predators consume prey containing MPs, magnifying effects across trophic levels	Zooplankton: Chameleon shrimp (*Praunus flexuosus*) and rockpool prawn (*Palaemon elegans*) [183]; Aquatic mammal: Spotted seal (*Phoca largha*) [184], Fish-eating killer whales [185]
Respiratory stress: Impaired respiratory functions	MPs clog respiratory surfaces like gills, reducing oxygen uptake	Sponge: Mediterranean sponge (*Petrosia* *ficiformis*) [186]; Fish: Dorab wolf-herring (*Chirocentrus dorab*) [186], Butterfish (*Drepane punctata*) [186]

### Impact of microplastics on the marine environment and organisms

Plastic pollution is one of the most pervasive and persistent threats to the global environment, particularly to marine ecosystems that cover over 70% of the Earth’s surface and support rich biodiversity [43]. Previous studies have documented the adverse effects of MPs and NPs on freshwater and marine organisms [83]. It is estimated that at least 10% of plastic waste enters the marine environment, causing severe global plastic pollution, especially from MPs [43]. MPs can also enter organisms through drinking water, posing risks to all forms of life. These particles act as carriers for toxic substances, including industrial and urban contaminants [84]. Microplastic-contaminated effluents are ultimately transferred into aquatic systems, where they are directly ingested by organisms. Furthermore, MPs indirectly threaten aquatic ecosystems by adsorbing other water pollutants [43].

The excessive discharge of billions of tons of plastic waste annually, from domestic to industrial sources, contributes to the accumulation of degraded MPs in aquatic systems. These contaminants not only pollute ecosystems but also infiltrate food chains [21]. Marine ecosystems, which harbor the largest biological diversity on Earth, rely on this biodiversity to maintain essential ecosystem functions such as biological production, habitat creation, nutrient cycling, oxygen production, carbon sequestration, and other vital services that support human well-being [85]. MPs and NPs can be absorbed by phytoplankton, consumed by animals, and accumulated within their bodies. Once these particles enter the food chain, they are transferred to higher organisms, magnifying concentrations through trophic transfer [86–88]. The widespread presence of MPs, particularly in marine environments, and their small size make them easily ingested by marine organisms, thereby increasing their entry into the food chain and impacting marine biota, such as phytoplankton [87–89].

MPs pose a growing threat to marine ecosystems and organisms. The risks associated with microplastic ingestion stem not only from the material itself but also from its ability to absorb and concentrate environmental contaminants, which are subsequently transferred through food chains. MPs are now recognized as emerging pollutants, capable of infiltrating even the most pristine areas of the planet, including the deep sea and polar regions [90]. Ingested MPs cause a range of toxic effects in aquatic organisms, including physical and chemical damage. Chemical effects may lead to endocrine disruptions, hepatic stress, oxidative stress, altered metabolic parameters, reduced enzyme activity, and cellular necrosis [91] (Table 1).

Long-term exposure to MPs has been shown to compromise growth, reproduction, and population survival [92]. Additionally, ingestion of MPs causes physical harm to smaller organisms by blockage of the digestive system, gills, and other organs [93]. Hence, plastic debris, now recognized as an emerging pollutant, represents a significant threat to marine biodiversity globally [94]. Various marine organisms, including phytoplankton, zooplankton, invertebrates, mollusks, corals, crustaceans, and fish, are affected by the ingestion of MPs, disrupting marine life and ecosystems [5,94].

### Plankton

Phytoplankton are essential primary producers in aquatic ecosystems, but they are vulnerable to microplastic contamination, which can have cascading effects on aquatic food webs [87,88]. MPs negatively impact biological parameters such as growth, chlorophyll content, photosynthesis, and oxygen production [5,95]. Zooplankton, as secondary producers, play a crucial role in marine food webs and biogeochemical cycling. Among zooplankton, copepods are highly valued as live feed for marine fish and shellfish larvae [5,88,96]. Research indicates that filter-feeding copepods, bivalves, and decapod larvae can ingest MPs [87,88,96]. Algal feeding by copepods has been shown to significantly reduce microplastic levels but can lead to smaller eggs with decreased hatching and survival rates [5,88,96].

### Crustaceans

Aquatic crustaceans such as shrimps, prawns, crabs, lobsters, and crayfish play dual roles as predators and prey in the food web, contributing to energy transfer [97]. They feed on plants, algae, and smaller organisms while serving as food sources for other species. MPs and NPs can enter shrimps, prawns, crabs, lobsters, crayfish, and other crustaceans through their gills and mouths, accumulating in internal organs. This accumulation negatively affects innate immunity, survival rates, tissue integrity, gut microbiota, and oxidative stress levels [83].

MPs have adverse effects on the feeding, swimming, grazing, and defense behaviors of shrimps, prawns, crabs, lobsters, and crayfish [97]. When coexisting with other contaminants, these effects are exacerbated, particularly for primary MPs, which reduce feeding efficiency more than secondary MPs [98]. Additionally, microplastic exposure impairs physiological functions such as oxidative stress regulation, metabolism, respiration, fecundity, offspring development, and enzyme activity [97]. Specific studies highlight the harm to shrimp gills, including structural damage, reduced oxygen intake, and increased infection risk [99].

### Corals

The impacts of macroplastics, MPs, and NPs on corals and reef ecosystems have gained significant attention. MPs pose a sustainability challenge with wide-ranging effects on marine ecosystems [28,100]. Coral reefs, formed by coral polyps within calcium carbonate skeletons, are biodiversity hotspots that support fisheries, tourism, and coastal protection. They also regulate tidal effects, generate oxygen, and absorb atmospheric carbon dioxide [101].

Microplastic ingestion can cause coral bleaching, necrosis, and zooxanthellae release [27,100]. Laboratory experiments have shown that microplastic ingestion inhibits food intake and reduces the growth rates of cold-water corals such as *Lophelia pertusa* [90]. Corals exhibit various responses to microplastic exposure, including attachment of particles to tentacles, ingestion, mucus production, and overgrowth [27,102]. Studies have found that 5.7% of ingested particles remain in the coral digestive system for at least 24 hours, potentially affecting energy balance, pollutant toxicity, and trophic transfer [103].

### Other invertebrates

Microplastic ingestion in invertebrates diminishes both somatic and reproductive growth, likely due to the diversion of energy from growth and reproduction processes to maintenance mechanisms of other physiological processes [104]. For example, the antioxidant system, which counteracts oxidative damage and restores redox homeostasis following pollution exposure, utilizes more energy to repair oxidative damage, resulting in less energy available for growth and reproduction [104]. A significant environmental risk posed by MPs is their resemblance to food, which can obstruct the digestive systems of marine organisms and impair functionality [105]. Deposit and detritus feeders, such as amphipods, are particularly susceptible to microplastic exposure as they may mistake them for natural food sources and become primary consumers of MPs [106].

Marine invertebrates with various feeding methods, including mussels (filter feeders), lugworms (deposit feeders), and sea cucumbers (detritivores), have been documented to ingest MPs due to their particle size similarity to plankton [105]. Microplastic exposure has shown both lethal and sublethal effects, including physical damage, toxicity, and behavioral disorders across diverse organisms, from invertebrates to birds and mammals [106].

Mollusks, highly nutritious and economically valuable, are particularly vulnerable to microplastic contamination. As typical filter feeders, bivalves constantly filter microbes and organic matter from their surroundings, potentially accumulating MPs. Mollusks are a potential source of human microplastic intake [5]. MPs in mollusks often consist of fibers, fragments, films, and pellets, with fibers accounting for more than 50% of microplastic types in freshwater and coastal environments [71]. Higher microplastic concentrations in mollusks suggest trophic transfer within the food web [107].

### Seagrass

Seagrass meadows offer numerous ecosystem services, including serving as spawning grounds for various fish species, facilitating carbon uptake and storage, and providing coastal protection. A key aspect of these services is their ability to create a three-dimensional habitat that reduces water velocity, promotes sedimentation, and traps particles [108].

The issue of microplastic pollution in marine environments has garnered increasing attention. Seagrasses, among the most productive shallow-water ecosystems, support a diverse range of fish and invertebrates [109]. However, there is a notable risk of MP accumulation in seagrass beds, potentially exceeding that in unvegetated marine areas [110]. For instance, studies have shown that MPs adhere to the surfaces of suspended seaweeds, such as *Fucus vesiculosus*, which are then ingested by grazing gastropods [111]. This provides the first evidence of seaweeds acting as an efficient pathway for transferring MPs from water to marine benthic herbivores [5].

Coastal environments located in intertidal and subtidal zones that store carbon in the soil and sediments of plants such as mangroves, salt marshes, and seagrasses, called blue carbon ecosystems, are significant sinks for MPs [112]. The friction caused by mangrove roots and seagrass leaves slows water flow, extending the residence time of external carbon sources and facilitating particle sedimentation [112]. These ecosystems trap MPs alongside particulate organic carbon (POC) from sources such as drifting macroalgae, terrestrial organic debris, and plant material [112].

Nevertheless, the accumulation of MPs in seagrass meadows and mangrove forests could harm vegetation growth and biodiversity in these sensitive ecosystems [113]. MPs in sediments and benthic organisms of seagrass beds may alter trophic interactions and ecosystem health. Ingested MPs can elicit biological responses through both physical and chemical mechanisms, providing potential pathways for persistent organic pollutants (POPs) to enter the food web [109]. For example, MPs can act as vectors for POPs by absorbing these toxic chemicals onto their surfaces from the surrounding environment [109]. POPs, such as PCBs, dioxins, and pesticides, are hydrophobic and tend to attach to plastic particles due to their chemical affinity for nonpolar substances [114]. When marine organisms such as seagrass ingest MPs, the POPs can desorb into their tissues, bioaccumulating and causing toxicity. This dual threat of physical harm from the MPs themselves and chemical harm from the attached POPs exacerbates the environmental and health risks associated with plastic pollution in marine ecosystems [109,114]. Moreover, MPs could spread from seagrass through the food web via trophic transfer, exacerbating their ecological impacts [93].

### Fishes

MPs are widely recognized as accumulating in freshwater and marine ecosystems, posing toxicological risks to aquatic organisms, particularly fish [115]. Exposure to environmentally relevant MPs has been linked to behavioral and histological changes in fish due to coping mechanisms [116]. Particles smaller than 500 nm can provoke immune responses, disrupt metabolism, alter gut microbiota, and even impair brain function [117]. Once ingested, MPs accumulate in fish tissues, as no mechanisms exist to excrete polymeric waste [115].

Studies combining electrophysiological and behavioral observations have revealed that microplastic fibers significantly impact fish, increasing coughing rates, reducing daily food intake, and stimulating excessive mucus secretion during ventilation, feeding, and swimming [116]. These changes highlight the pressing need to address microplastic contamination, particularly in fish, which serve as vital protein sources for humans.

Microplastic exposure can cause oxidative stress, immune dysfunction, and alterations in gene expression and antioxidant status in fish, leading to neurotoxicity, growth retardation, and behavioral abnormalities [118]. For example, zebrafish exposed to Petit Bourg MPs exhibited decreased reproductive success [91]. Furthermore, microplastic-contaminated fish can act as vectors for heavy metals, potentially causing diseases such as diabetes, cancer, and Alzheimer’s in humans [119].

Ingested MPs may also block the digestive tract, suppress appetite, and disrupt normal energy transfer, exposing internal organs to these harmful particles [118,120]. Studies on *Danio rerio* have frequently reported oxidative stress, reduced mobility, and reproductive organ damage due to microplastic exposure [118]. Oxidative stress induced by MPs also disrupts fish metabolism, impacting lipid and carbohydrate processes [120]. Additionally, microplastic uptake can trigger inflammatory responses in the fish intestine, evidenced by increased leukocyte infiltration into damaged tissues [121]. Growth reductions in fish exposed to MPs have been observed, highlighting potential long-term ecological consequences [122]. Alarmingly, it is predicted that oceanic plastic mass will surpass fish biomass by 2050, underscoring the urgent need for mitigation strategies [122].

Moreover, studies have found microplastic particles embedded in tissues surrounding the brain, affecting neurological functions [123]. Biomarker assessments reveal that MPs cause DNA damage, neurotoxicity, and oxidative stress in fish, making them more susceptible to diseases [124,125]. These findings stress the critical importance of addressing microplastic contamination, not only to protect aquatic ecosystems but also to mitigate risks to human health through the consumption of contaminated fish.

### The impact of microplastics on aquaculture

Aquaculture plays a significant role in providing aquatic products and dietary protein for many households. However, like other food sectors, it faces considerable challenges, including unfavorable climatic events, environmental stressors, and contaminants. Among these, MPs have emerged as critical pollutants in aquaculture, evidenced by their presence in cultured fish, fishmeal, and aquafeed [187]. The extensive use of plastic equipment in aquaculture contributes to high MP contamination levels in the environment. MPs are readily ingested by aquatic organisms, transferring through the food web to higher trophic levels, including humans [20]. Studies show that approximately 60% of farmed aquaculture species and 80% of wild-caught marine species have demonstrated an ability to ingest MP debris [188].

Major sources of MPs in aquaculture include wastewater from treatment plants, aquaculture facilities, fishing gear, PPE, fishmeal, and feedstuff [187]. Additionally, aquatic activities such as fisheries, shipping, and tourism are potential contributors to MP pollution. Equipment used in aquaculture, such as cages, ropes, floats, and nets, often suffers damage from severe weather, releasing MPs into the environment. Cleaning activities to remove biofouling organisms also generate MP fibers [189]. Offshore aquaculture systems, particularly mariculture, are highly vulnerable to MPs due to the accumulation and availability of these particles, posing significant threats to marine species [190].

MP contamination in aquaculture environments is classified into two categories: primary MPs (manufactured as micro-sized particles for commercial applications) and secondary MPs (formed from the degradation of larger plastics due to environmental factors) [191]. Closed and semi-closed freshwater systems exhibit particularly high MP accumulation. For example, in Asian swamp eel (*Monopterus albus*) aquaculture, MP concentrations in post-cultured water were significantly higher than in pre-cultured water [192]. Aquaculture products, including fish, shrimp, crabs, and mussels, accumulate MPs through their digestive systems, gills, and skin, often at higher levels than wild species. For instance, cultured mussels were found to have 1.4–1.7 times more MPs than their wild counterparts [117].

The physical and chemical effects of MPs on aquatic organisms vary based on their size, shape, and chemical composition. MPs cause oxidative stress, disrupt growth and reproduction, and lead to economic losses in aquaculture [117]. MPs also release additives and contaminants such as heavy metals and antibacterial agents, potentially altering the ecological balance of aquaculture systems [193]. Exposure to MPs in organisms such as *Oreochromis urolepis* larvae causes severe damage to intestinal structures and cellular patterns, affecting overall health [194].

MPs were also detected in the gastrointestinal tracts and gills of cultured common carp (*Cyprinus carpio*), with plastic polymers such as PP and PS being the most common [195]. Hence, plastic mitigation efforts in aquaculture are increasingly gaining attention as the industry seeks to reduce its environmental impact. Key initiatives include developing biodegradable and environmentally friendly alternatives to traditional plastic gear, such as nets, ropes, and buoys, which often degrade into harmful MPs [196]. Many aquaculture operators are adopting closed-loop systems to minimize plastic waste and prevent its release into marine environments [196]. Efforts also focus on recycling and repurposing old equipment to extend its lifecycle and reduce dependency on primary plastic [196].

### The impact of microplastics on human health

Microplastic exposure poses potential risks to human health, including respiratory and digestive problems, disrupted endocrine functions, and increased risks of obesity and diabetes [197]. Studies reveal their presence in various food and beverages, including drinking water, honey, and canned goods, highlighting their ubiquity in the human diet [198]. However, MPs can enter the human body primarily through ingestion of contaminated seafood, drinking water, and food packaged in plastic [29,199]. Once ingested, MPs may accumulate in the digestive tract and other organs, triggering inflammatory responses and oxidative stress [199]. Chronic exposure to MPs has been linked to toxicity, carcinogenicity, and disruptions to the intestinal microbiome, leading to gastrointestinal issues [199,200]. A study in a mouse model revealed that NPs released from PE bags caused hematological aberration, such as a significant reduction of RBC count and a remarkable enhancement of WBC count, especially the percentage of monocytes [201]. In addition, it caused histological damage to the intestine, heart, lung, kidney, and liver by disruption of muscle layers, fibrosis, and infiltration with foam cells [201].

MPs can also enter the body via dermal exposure, such as through synthetic textiles or open wounds [202]. These particles carry harmful additives and contaminants, including heavy metals and organic pollutants, which can exacerbate their toxic effects. The potential for MPs to cross biological barriers, such as the gastrointestinal epithelium, placenta, and blood–brain barrier, underscores their systemic impact [203]. Airborne MPs from agricultural practices, industrial emissions, and marine aerosols contribute to respiratory problems and autoimmune diseases [204]. Long-term exposure to MPs disrupts cellular processes by increasing oxidative stress, ROS accumulation, and mitochondrial dysfunction, leading to cytotoxicity [46]. Additionally, the endocrine-disrupting properties of MP additives can lead to hormonal imbalances, reproductive issues, and an increased risk of certain cancers [205]. However, clinical data on the effects of MPs on human health are still inadequate. Tracking individuals with different exposure levels to MPs can provide critical insights into the potential links between MPs and adverse health outcomes. These studies can help identify biomarkers of exposure and establish dose-response relationships, which are essential for assessing risks and setting safety thresholds. Furthermore, longitudinal research can reveal the long-term effects of chronic exposure to MPs, which is particularly relevant given the pervasive presence of MPs in food, water, and air. By filling these knowledge gaps, human clinical studies can inform public health guidelines, regulatory policies, and strategies to mitigate exposure, ensuring better protection of human health in a plastic-polluted world.

### Degradation of plastics

Conventional plastics are highly resistant to degradation, with lifespans estimated to range from hundreds to thousands of years depending on their properties and the surrounding environmental conditions [206]. Plastic degradation varies across environments. In marine ecosystems, photodegradation predominates in surface waters, while biodegradation by microorganisms becomes significant in the aphotic zone [20,55]. While plastics degrade slowly through biological and abiotic processes, environmental weathering contributes to their breakdown over time. Plastics buried in soil degrade more rapidly than those in ocean sediments due to particle collisions and other physical processes [45]. Comprehensive reviews of the degradation mechanisms for various plastics have been conducted [15] (Table 2).

### Biotic degradation of plastics

Biotic degradation occurs when organisms break down plastics either physically (e.g., biting, chewing, or digestive fragmentation) or biologically (e.g., biochemical processes) [15,207]. Microorganisms such as bacteria, fungi, and insects play a significant role in this process [208]. Plastics can be classified as hydrolyzable or nonhydrolyzable based on their chemical composition. Hydrolysable plastics, such as polyesters and PAs, are more susceptible to degradation due to their ester or amide groups, which can be targeted by extracellular hydrolases [15]. PET, commonly used in beverage bottles, has been the focus of research, with studies exploring its biodegradation mechanisms [209]. Nonhydrolysable plastics, including PE, PP, PS, and PVC, are more resistant to degradation but may be broken down by enzymes involved in lignin degradation [15,210].

**Table 2.** Microplastic degradation in the marine environment.

Plastic type	Degradation rate (years)	Key microorganisms involved	Environmental factors in marine ecosystems	Degradation methods	References
PE	100–1,000+	*Alcanivorax *spp*., Rhodococcus *spp*., Pseudomonas *spp*., Pseudalkalibacillus *sp*., Vibrio alginolyticus, Microbulbifer pacificus, Bacillus subtilis*	Low UV penetration, oxygen scarcity, and biofilm formation	Photodegradation, microbial degradation	[50,211–219]
PP	20–30+	*Vibrio *spp*.,* *Bacillus amyloliquefaciens., Bacillus *spp*., Pseudomonas *spp*., Pseudoalteromonas *spp.	Saltwater abrasion and UV exposure at surface	Oxidative degradation and microbial activity	[215–220]
PVC	50–100+	*Halomonas *spp*., Bacillus subtilis*	Chlorine content resists microbial action, limited UV impact	Hydrolytic and oxidative processes	[215–217,219,221,222]
PS	500–1,000+	*Pseudomonas *spp*., Achromobacter xylosoxidans, Pseudoalteromonas *spp.	Slow biofilm colonization, low microbial affinity	Microbial degradation with pre-treatment	[215–217,219,220]
PET	450+	*Vibrio alginolyticus, Pseudoalteromonas caenipelagi, Microbulbifer pacificus, Pseudomonas marincola, Bacillus subtilis, Ideonella sakaiensis, Halomonas *spp*.*	Stable in saline environments, susceptible to enzymatic breakdown	Enzymatic hydrolysis and photodegradation	[50,215–217,219,223]
PLA	Rare in marine (depends on conditions)	*Vibrio *spp*., Bacillus *spp*.*	Requires elevated temperature and oxygen not typical in oceans	Hydrolysis under specific conditions	[215,216,219,224,225]

### Abiotic degradation of plastics

Abiotic degradation refers to the changes in the physical or chemical properties of plastics due to nonliving factors such as light, temperature, air, water, and mechanical forces [226]. This process is primarily driven by exposure to environmental elements such as UV radiation, heat, moisture, and various chemical agents [227,228]. A few key mechanisms of abiotic degradation of plastics are photodegradation, thermal degradation, oxidative degradation, chemical degradation, and mechanical degradation [227].

Unlike biotic gradation, abiotic degradation does not fully eliminate plastic pollution. Instead, it transforms it into smaller fragments that remain environmentally persistent, worsening ecological and health concerns [227]. Moreover, the rate and extent of abiotic degradation depend on various factors, including the type of plastic, environmental conditions, and the presence of additives in the plastic material. For instance, certain plastics like PE and PP degrade very slowly, while others, like polylactic acid (PLA), degrade more quickly under specific conditions [227,228]. However, abiotic and biotic processes of degradation of MPs are deeply interconnected, shaping ecosystems and influencing one another in dynamic ways [229]. Abiotic factors create the conditions that influence the distribution, growth, and behavior of microorganisms involved in the biotic degradation of MPs [229]. Conversely, biotic processes, such as microbial growth, decomposition, and animal activities, actively modify abiotic factors [229]. Therefore, understanding both biotic and abiotic degradation processes is essential for developing strategies to mitigate plastic pollution and designing more environmentally friendly plastic materials.

### Photodegradation of plastics

Photodegradation, primarily initiated by solar UV irradiation, is a key process for plastic degradation in the environment. UV radiation generates free radicals that initiate degradation reactions [230]. While PE is resistant to photodegradation due to a lack of chromophores, impurities or defects can act as chromophores, facilitating degradation [231]. PVC, under certain conditions, can degrade into smaller molecules, such as ketones and alcohols [46].

### Thermal degradation of plastics

Thermal degradation occurs when plastics are exposed to elevated temperatures, leading to thermos-oxidative reactions. This process generates radicals, which propagate degradation until energy input ceases or stable products form [15]. Temperature and oxygen availability significantly influence thermal degradation [208].

### Oxidative degradation of plastics

Oxidative degradation plays a significant role in the abiotic breakdown of plastics. This process involves the reaction of oxygen in the atmosphere with the plastic material, resulting in the formation of weak spots within the polymer matrix [227,228]. Over time, this reaction leads to structural weakening and fragmentation. While these processes contribute to the disintegration of plastics, the outcome is often the formation of MPs that persist in the environment [227,228].

### Chemical degradation of plastics

Chemical degradation arises from interactions with pollutants like ozone (O₃), sulfur dioxide (SO₂), nitrogen dioxide (NO₂), and volatile organic compounds (VOCs). These pollutants either directly attack plastics or catalyze radical formation through photochemical reactions, accelerating degradation [208,232]. In aquatic environments, factors such as pH and salinity can also influence plastic degradation and alter their interaction with other pollutants [230].

### Mechanical degradation of plastics

Mechanical degradation occurs due to external forces such as wind, waves, or abrasion with rocks and sands. Processes like freezing and thawing in aquatic environments also contribute to degradation. Domestic washing and garment wear release microplastic fibers into the environment, posing additional challenges [233,234].

## Recommendations

Further research is necessary to understand the health impacts of microplastic consumption on marine organisms and their transfer through the food chain. Quantitative studies are needed to explore how MPs facilitate the transfer of toxic chemicals to marine life and, subsequently, to humans. To encourage innovations that reduce plastic use and develop alternatives, we must raise public awareness. Effective waste management policies, including recycling and biodegradation strategies, are essential to minimize the entry of plastics into ecosystems and prevent future threats. In addition, future research should be focused on the synergistic effects of MPs with other pollutants, such as heavy metals and pesticides, in marine environments. These interactions may amplify the ecological and toxicological impacts of MPs, posing compounded risks to marine organisms and ecosystems. Looking into these combined effects will help us learn more about how complex pollutants interact with each other, help us make better risk assessments, and inspire the creation of more all-encompassing ways to reduce many problems caused by marine pollution.

## Conclusion

In conclusion, MPs represent a severe and persistent threat to the marine environment due to their widespread sources and detrimental impacts. Predominantly entering from external sources such as plastic waste and human activities, MPs accumulate in marine ecosystems, adversely affecting marine organisms, ecosystems, aquaculture, and even human health. Their interactions with marine life result in significant physiological, behavioral, and reproductive harm. Moreover, the slow degradation of MPs exacerbates their environmental persistence. Therefore, understanding their sources, effects, and degradation processes is critical for developing effective strategies to mitigate their harmful impacts and protect marine ecosystems.

## List of abbreviations

As, arsenic; Cd, cadmium; DDTs, dichlorodiphenyltrichloroethane; MPs, microplastics; NO₂, nitrogen dioxide; NPs, nanoplastics; O₃, ozone; PA, polyamide; Pb, lead; PCBs, polychlorinated biphenyls; PE, polyethylene; PET, polyethylene terephthalate; PLA, polylactic acid; PMMA, polymethyl methacrylate; POC, particulate organic carbon; POPs, persistent organic pollutants; PP, polypropylene; PPE, personal protective equipment; PS, polystyrene; PVC, polyvinyl chloride; RBC, red blood cell; SO₂, sulfur dioxide; UV, ultraviolet; VOCs, volatile organic compounds; WBC, white blood cell; WWTPs, wastewater treatment plants.
